# Molecular Modelling Guided Modulation of Molecular Shape and Charge for Design of Smart Self-Assembled Polymeric Drug Transporters

**DOI:** 10.3390/pharmaceutics13020141

**Published:** 2021-01-22

**Authors:** Sousa Javan Nikkhah, Damien Thompson

**Affiliations:** Department of Physics, Bernal Institute, University of Limerick, V94 T9PX Limerick, Ireland; damien.thompson@ul.ie

**Keywords:** dendritic polymers, polyelectrolytes, cyclic polymers, self-assembly, smart drug nanocarriers, molecular modeling

## Abstract

Nanomedicine employs molecular materials for prevention and treatment of disease. Recently, smart nanoparticle (NP)-based drug delivery systems were developed for the advanced transport of drug molecules. Rationally engineered organic and inorganic NP platforms hold the promise of improving drug targeting, solubility, prolonged circulation, and tissue penetration. However, despite great progress in the synthesis of NP building blocks, more interdisciplinary research is needed to understand their self-assembly and optimize their performance as smart nanocarriers. Multi-scale modeling and simulations provide a valuable ally to experiment by mapping the potential energy landscape of self-assembly, translocation, and delivery of smart drug-loaded NPs. Here, we highlight key recent advances to illustrate the concepts, methods, and applications of smart polymer-based NP drug delivery. We summarize the key design principles emerging for advanced multifunctional polymer topologies, illustrating how the unusual architecture and chemistry of dendritic polymers, self-assembling polyelectrolytes and cyclic polymers can provide exceptional drug delivery platforms. We provide a roadmap outlining the opportunities and challenges for the effective use of predictive multiscale molecular modeling techniques to accelerate the development of smart polymer-based drug delivery systems.

## 1. Introduction

The efficacy of Active Pharmaceutical Ingredients (API) is often hampered by low aqueous solubility and short residence times in the body [1]. Conventional drug delivery formulations such as tablets, capsules, pills, solutions, powders, suspensions, injectables, lotions, creams, pastes, and, etc., have contributed greatly to the treatment of disease. The impetus for smart delivery methods has escalated due to several factors. These include low efficacy, the difficulty in keeping the drug levels within a desired range, minimising side effects and toxicity, and the emergence of specific biological therapeutics [2,3]. Research into newer smart drug delivery systems is being carried out using liposomes, nanoparticles, niosomes, transdermal drug delivery, implants, microencapsulation, polymers, aerosols, etc. These studies show their potential for higher bioavailability, less side effects, less total amount of required drug, more patient compliance, less tissue damage and lower price, compared to the conventional methods [4].

One promising approach to improve the physicochemical properties of APIs is to engineer smart nanoparticle (NP)-based drug delivery systems [5,6,7]. The ability of the NPs to enter the cell is determined by both physicochemical parameters and biological barriers. Due to the high surface area to volume ratio (small size), they are able to penetrate the cell membrane and deliver the drug inside the cell [8]. NP drug carriers can improve drug solubility, biocompatibility and half-life, and can reduce dosage frequency and side effects by transporting drugs to specific and targeted sites. This novel class of multifunctional drug carrier platform can potentially combine diagnostic agents and targeted multidrug therapies together in a single system [9,10,11,12,13].

Smart nanostructured systems can be categorized into two main groups of organic and inorganic nanocarriers, the physiochemical characteristics of which can be tuned by changing their compositions, shape, dimension, and surface properties [14,15]. The organic group includes polymer micelles [16], liposomes [17,18,19,20], dendrimers [21,22,23], glycopeptides [24,25] and protein assemblies [26,27], while carbon-based nanomaterials [28,29], gold nanoparticles [30,31], silver nanoparticles [32,33], porous silica-based nanomaterials [34,35,36], and various metal-based quantum dots [37,38] belong to the inorganic group.

The promise and potential of NPs has been discussed in numerous review articles covering their history, advances, advantages, potentials and limitations [39,40,41,42,43,44,45,46,47,48,49,50]. Research articles have reported valuable knowledge on their intracellular transport [51,52], targeted drug delivery to tumors [53], internalization and cellular uptake mechanism (localization of intracellular nanoparticles) [54,55], interfacial biophysicochemical interactions [56], mechanisms of nanoparticle endocytosis [51,57], cellular excretion and degradation of nanoparticles [58] and toxicity (cytotoxicity and immunogenicity) [12,59]. The amount of often-conflicting data makes it difficult to draw general conclusions about how to produce particles for optimal drug delivery. There is still a lot to learn about NP-based drug delivery mechanisms in order to interpret data from in vitro studies and to improve the in vivo use of the particles. Thus, understanding the whole cellular process induced by NPs would provide a rational basis for materials design, tuning their interaction with the cell membrane, and improving their uptake by cells [60,61].

The in vivo availability and efficacy of drug delivery systems are mainly determined by their pharmacokinetics. The pharmacokinetic parameters of drug delivery systems are size, shape, composition, administration route, and surface modification. All these parameters also influence pharmacodynamics. While the main advantage of the smart NPs drug delivery systems as compared to the conventional ones is having controllable pharmacokinetic parameters [8], it can be very difficult to predict *a priori* the material performance given the broad range and interdependence of the parameters.

Understanding how the smart NPs form and perform becomes crucial for the future development of efficient drug delivery. However, there is still a lack of knowledge about the interactions and processes in mediated drug transport due to the short time and length scales at which nanocarriers operate, which can rarely be detected by experiments alone. Appropriately benchmarked and parameterized computer simulation methods can supply the necessary molecular details to build a deeper understanding of how the API is encapsulated and transported [13,62].

Different molecular modeling approaches scaling from ab initio quantum mechanics (QM) to classical molecular mechanics (MM), molecular dynamics (MD), Monte Carlo (MC) methods and out to mesoscale (MS) techniques cover the broad range of both length and time scales to design a complete drug delivery system [63,64,65,66,67] (see Table 1 for more details of the simulation methods). QM methods can provide exceptional accuracy by obtaining the electron distribution of any molecular system but with practical size limitations of few hundred atoms due to sharply rising computational cost. The MD level can capture all non-covalent interactions at atomic resolution for systems of, typically, a few hundred thousand atoms with microsecond sampling times [68]. However, many critical problems in this field still require time and length scales far beyond atomistic MD, which can be modelled by mesoscale simulations that have been appropriately parametrized using atomistic simulations of the component building blocks and interfaces. Mesoscopic simulations are performed using a coarse-grained molecular model formed by particles which are related to a group of atoms in the corresponding atomistic structure [13]. Coarse-grained molecular dynamics (GCMD) [69], MARTINI [70], and dissipative particle dynamics (DPD) [71,72,73] are some of the most popular mesoscopic simulation techniques that have been applied to study the self-assembly of NPs from polymeric building blocks, providing valuable insights and design principles for the rational engineering of novel drug delivery systems [13].

**Figure 1:**
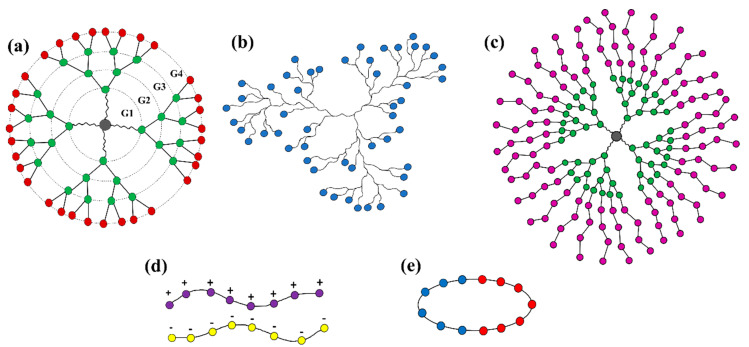
Schematic structure of (**a**) “star burst” dendrimer (of generation 4, G4), (**b**) hyperbranched polymer, (**c**) dendrimer multi-arm copolymer, (**d**) polyelectrolyte (polycation and polyanion) chains, and (**e**) cyclic polymer.

The present review aims to highlight the key common features of recent successful modeling-led investigations into the use of organic polymer-based NPs in drug delivery. We discuss the advanced topologies and chemistries of functional polymers including dendrimers, hyperbranched polymers, cyclic polymer and polyelectrolyte-based micelles (Figure 1) that can self-assemble into multifunctional smart drug carriers.

## 2. Polymer-Based Smart Nanocarriers

Organic nanocarriers are generally characterized by their tunable morphology, colloidal stability, relatively large size, high biocompatibility and improved drug loading capacity, which make them suitable for transporting a wide variety of drugs [84,85,86,87,88,89,90,91,92]. However, it is very important to make sure that the polymeric drug nanocarriers are safe and do not trigger cytotoxicity at the tissue level. Safe, smart NPs formulations for drug delivery must be biocompatible and have low immunogenicity; thus, they should be carefully designed and evaluated [93].

They can be divided into two main categories: (1) nanostructures that form through the self-assembly of short polymer chains and (2) synthesized large polymers (such as the dendrimers, hyperbranched polymers, chemical nanogels). The latest generation of smart supramolecular nanocarrier often is made by combining the two types [94,95,96]. However, the common point between these smart NPs categories is that they form through self-assembly of smart polymers. These so-called smart polymers have been defined as polymers that can undergo physical and structural conformational changes/rearrangements in response to mild changes in their surrounding environment, categorized as thermo-, pH-, electro- and magneto-responsive polymers [97]. In the following, we discuss the main characteristics of dendritic, cyclic polymers and polyelectrolytes, and critically assess recent efforts to predict their NP drug carrier potential via modelling.

### 2.1. Dendritic Polymers

Dendritic polymers are branched polymers with useful encapsulation properties including high degree of branching, high density of terminal functional groups, and nanometric size [98]. There are two main groups of dendritic polymers: dendrimers and hyperbranched polymers (see Figure 1a,b). Dendrimers are monodisperse polymers with perfectly branched architectures that are known for their well-organized structures, versatility in drug delivery and high functionality. Their potential abilities to physically entrap or conjugate high molecular weight molecules have been proven. Dendrimers could also be decorated to make them smart enough to carry the drug to the desired locus and release it in a controlled manner [99,100,101,102,103].

The introduction of stimuli responsive functionality on dendrimers allows the release of drugs in response to a specific trigger only. These triggers described below could be endogenous in nature (acid, enzyme, and redox potentials) or could be applied externally (light and temperature) [104]. **pH-responsive dendrimers:** Presence of ionizable functional groups such as amine and carboxylic acid on the surface or in the core of the dendrimer exhibit a pH-dependent release due to change of amphiphilicity of the system [105]. **Redox-responsive dendrimers**: The frequently used redox-responsive linkers for dendrimer–drug conjugate, such as disulfide bonds, diselenide or ditellurium bonds [106]. **Enzyme-responsive dendrimers:** Incorporation of drug molecules as the tail units and an enzyme substrate as the trigger in dendrimers, generating a prodrug unit that is triggered upon a single enzymatic cleavage. The enzymatic trigger commonly utilized is 38C2 antibody, penicillin-G-amidase or β-galactosidase [107]. **Temperature-sensitive dendrimers:** Modification of dendrimer surfaces with oligo- and poly(ethylene oxide)-based groups endows them with temperature-sensitive characteristics. There is an inverse relationship between aqueous solubility and temperature for temperature sensitivity functionalities. As temperature is increased, the degree of hydrogen bonding between the temperature sensitive moieties and water decreases, and this leads to phase separation. Lower critical solution temperature (LCST) is the phrase used to describe such phase transition [108,109]. **Light-responsive dendrimers:** The principle governing the release of drug from dendrimers using light as a stimulus is based on (i) the absorption of light by photosensitive ligands that would trigger configurational changes (e.g., *trans*-*cis* isomerization) and cause the release of the encapsulated drugs and (ii) the absorption of light by photosensitive ligands causing irreversible cleavage reactions. The most common photosensitive ligands for (i) are azobenzene derivatives and for (ii) are *o*-nitrobenzyl ether derivatives grafted on the surface of dendrimers [110].

Dendritic polymers have tuneable surface charge and chemical composition obtained by surface modification with charged moieties (such as amine-, carboxyl-, and acetyl-) [111] and by special functional side groups (such as isobutyramide and poly(ethylene glycol)) [112,113], which also makes them controllable in size (reported between 1 nm and 100 nm) and physiochemical properties [114]. Owing to these features, dendrimers have been used in the development of drug nanocarriers and many therapeutic and biomedical applications [115,116,117]. In Table 2, some clinical studies that have been done on dendritic polymer NPs as drug delivery systems are presented.

A complete dendrimer structure (Figure 1a) consists of an exterior multivalent (multi-site) surface with a multitude of potentially active or passive sites and the interior layers surround the core [118,119]. Higher-generation dendrimers have more branches. Depending on their hydrophobicity/hydrophilicity, drug molecules can occupy the vacant spaces (voids) within the interior layers, or else bind to dendrimer surface functional groups either physically or covalently [120,121,122].

Although the properties of dendrimers render them suitable as pharmaceutical excipients, they also present less advantageous properties, which may hinder their use, namely, their cytotoxicity, the limitation of incorporation of the drug into the dendrimer cavities, and the inability to control the rate of drug release. Furthermore, this type of polymer also presents high manufacturing costs and the need for a specialized workforce [123]. The new developments have allowed for increased industrial manufacturing efficiency and lowered production costs [124]. In order to reduce the cytotoxicity and to increase the space on the dendrimer cavity, numerous modifications have been proposed to the chemical structure of the dendrimers. These alterations make dendrimers more suitable for use as pharmaceutical excipients. Additionally, PEGylation of dendrimers increases their blood circulation time. The lack of control of the rate of drug release from the dendrimer can be avoided by covalent conjugation of the drug to the dendrimer surface. Drug release is then dependent on the cleavage of the dendrimer–drug linkage [114].

On the other hand, randomly branched polymers, also known as hyperbranched polymers, have advantages due to their low intrinsic viscosity, low tendency to chain entanglements, good solubility and high degree of branching (see Figure 1b), which have been exploited for development of smart nanocarriers for drug delivery [125,126]. In addition, the composition of the branching, linear, and terminal units of hyperbranched polymers can be engineered to be responsive to one or multiple stimuli, which leads to a significant degree of freedom in the molecular design of smart hyperbranched polymer-based nanocarriers for drug delivery [127].

Dendritic polymers have also shown great potential as building units for self-assembled NPs. Known generally as dendrimer multi-arm copolymers or hyperbranched multi-arm copolymers, the dendritic polymers used in self-assembly (Figure 1c) exhibit an amphiphilic structure with a hydrophobic (or hydrophilic) core and many hydrophilic (or hydrophobic) linear arms [57]. Many supramolecular aggregates with diverse structures and morphologies have been formed through self-assembly of amphiphilic dendritic polymers, including macroscopic tubes [133], physical gels [134], vesicles [135,136,137], spherical micelles [138,139,140,141], and honeycomb films [142], bridging atomic to macro space-time scales. Below, we select recent research work that illustrates how molecular modeling of dendritic polymers can guide the engineering of useful drug carriers by controlling supramolecular interactions and self-assembly.

**Figure 2:**
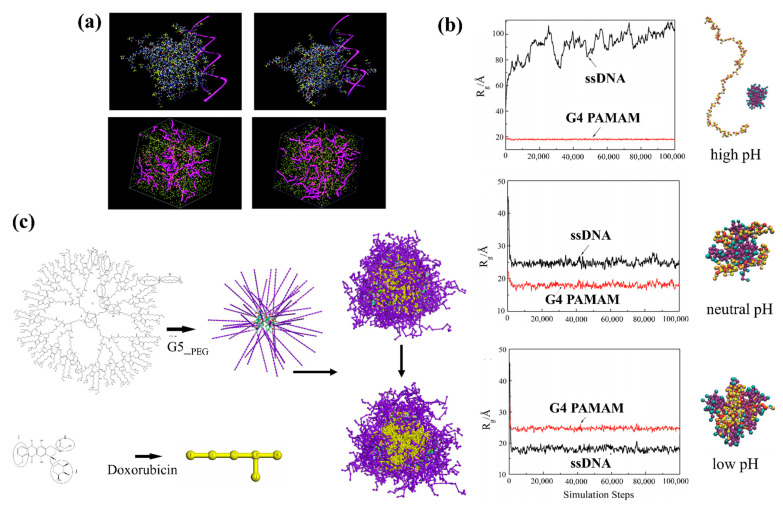
(**a**) Triethanolamine (TEA)-core dendrimer (**top left**), NH_3_-core dendrimer (**top right**) in complex with a small fragment of double-helix DNA (highlighted as a magenta ribbon). Mesoscale morphologies of the self-assembled systems between TEA-core dendrimers and DNA (**bottom left**) and NH_3_-core and DNA (**bottom right**) [143]. (**b**) Evolution of the radius of gyration (R_g_) of ssDNA and G4 PAMAM dendrimer at various pH values [144]. (**c**) Chemical structures of dendrimer and drug molecules in DPD simulation (**left**), their corresponding simplified beads in the coarse-gained models (**middle**), and the computed G5-PEG/DOX microspheres (**right**) [147].

Liu et al. [143] compared the interaction of triethanolamine (TEA) core and NH_3_ core polyamidoamine (PAMAM) dendrimers with DNA using atomistic molecular simulations in explicit solvent, at physiological ionic strength (0.15 M) and pH = 7.4. The TEA-core PAMAM showed open flexible conformations with voids localized within its interior shells, while the NH_3_-core structure showed more rigid conformation, with a more homogeneous distribution of the monomer units and voids throughout the entire molecule. The TEA-core dendrimers then showed a preference for binding the charged phosphate backbone of DNA to their outer branches during complex formation. The simulations showed that the more flexible dendrimer architecture could achieve conformational rearrangement of its amine to optimize induced-fit with DNA (see Figure 2a).

Su et al. [144] employed DPD simulations to study the complexation of the PAMAM dendrimer and short ssDNA molecules. They built the coarse-grained model of ssDNA model according to the wormlike chain (WLC) potential [145] and quantified the effects of pH, dendrimer generation, salt concentration, and dendrimer/ssDNA charge ratio on the structure of the ssDNA–PAMAM complexes. They found that the ssDNA molecules were significantly compacted by PAMAM dendrimers at neutral or low pH, with the most stable ssDNA–PAMAM complex observed at low pH (see Figure 2b). They suggested that the release of ssDNA from dendrimer could be modified by using different generations of dendrimer. They also pointed out that the charge ratio between PAMAM dendrimer and ssDNA can be used to tune the size and morphology of the self-assembled aggregates, which could increase the transfection efficiency of ssDNA molecules in dendrimer-based gene vectors.

PEG–polyester dendrimers are one of the most attractive dendrimers for in vivo drug delivery due to their biodegradability and solubility achieved by PEGylation, their low cytotoxicity, and long half-life in the circulation system [146]. The potential of polyester-PEG dendrimers for in vivo delivery of anti-cancer drug doxorubicin (DOX) was studied by Wen et al. [147] using DPD simulations to investigate the loading/release mechanism of DOX in generation 5 polyester-PEG (G5-PEG polyester) dendrimers. In each dendrimer molecule, units G1 to G4 consisted of aliphatic polyester blocks and the outermost G5 layer contained 56 PEG blocks (see Figure 2c).

They found four sequential transient stages during DOX encapsulation: (1) initial random distribution of all the components in the simulation box, (2) dispersion of DOX molecules in the G5-PEG dendritic microsphere, (3) core-shell microsphere growth by coalescence of the small core–shell dendritic microspheres, and (4) stabilization (see Figure 2c). According to their results, in their system that contained 12.5 and 2.5% (mass fraction) of G5-PEG and DOX, 16.7% of DOX molecules were loaded to the core–shell dendritic microspheres with a loading efficiency of 100%, which is close to the experiment results [113]. The simulation also confirmed that no DOX molecule was released from G5-PEG/DOX at pH 7.4 in the simulation temperature range, from 25 to 37 °C, which means temperature was not the major driver for drug release at pH 7.4. Furthermore, at pH 5, the formation of some pores on the surface of G5-PEG/DOX microspheres increased the exposure of DOX molecules to water but did not trigger release. The authors concluded that the protonation of G5-PEG may facilitate the drug release process but it is not the major factor governing rapid release.

**Figure 3:**
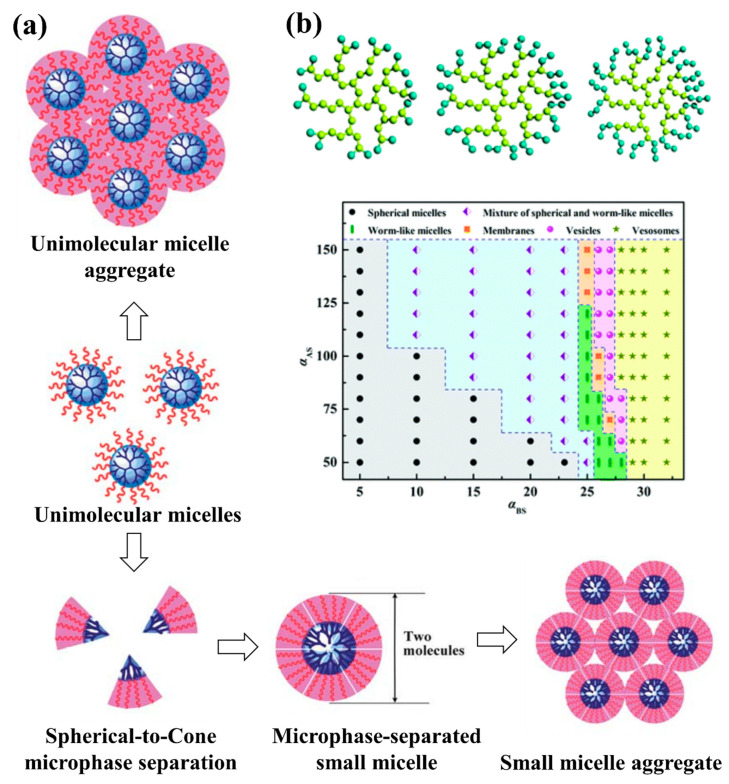
(**a**) Self-assembly mechanism of the microphase separated small micelle with a spherical-to-cone microphase separation [148] and inset (**b**) morphological phase diagram of aggregates formed from A_42_B_20_ in selective solvents as a function of the interaction parameters. A bead is hydrophobic and B bead is hydrophilic [149].

Modelling studies have provided useful insights to guide design of polymer-based NPs structures and morphologies through self-assembly of copolymers with different architectures, in order to create novel multifunctional platforms with promising application in drug delivery. One excellent example came from Wang et al. [148] in a systematic DPD simulation study of micelle formation from amphiphilic dendritic multi-arm copolymers in dilute solution. The authors simulated three models for dendritic multi-arm copolymers with different lengths of the arms and one model for hyperbranched multi-arm copolymers. Two kinds of mechanisms, namely the unimolecular micelle aggregate mechanism and the small micelle aggregate mechanism, were found to support the formation of large multimolecular micelles from the dendritic multi-arm copolymers (see Figure 3a). For the unimolecular micelle aggregate mechanism, the dendritic multi-arm copolymers first form the unimolecular micelles, and then the unimolecular micelles further aggregate into large micelles without microphase separations. For the small micelle aggregate mechanism, the dendritic multi-arm copolymers first self-assemble into microphase-separated small micelles, and then the small micelles further aggregate into large ones. These simulation results supported the experiments very well and extended general understanding of the micellization processes of dendritic multi-arm copolymers.

Amphiphilic hyperbranched multi-arm copolymers were also studied by Tan et al. [149] using DPD simulations. Their comprehensive study of the self-assembly of amphiphilic hyperbranched multi-arm copolymers with different hydrophilic fractions in various solvents resulted in three morphological phase diagrams. A variety of morphologies, ranging from spherical micelles and worm-like micelles to membranes and vesicles, were obtained (see Figure 3b). They also discovered several novel structures, such as aggregates of spherical and worm-like micelles, vesosomes and helical micelles, generated during self-assembly of amphiphilic hyperbranched multi-arm copolymers. Their results extend knowledge of the self-assembly of amphiphilic hyperbranched multi-arm copolymers, especially on the control of supramolecular interactions for the realization of novel self-assemblies.

**Figure 4:**
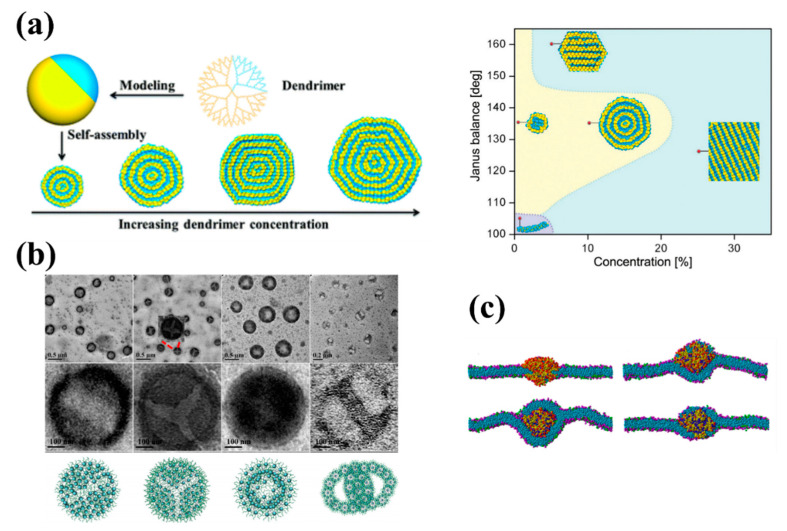
(**a**) Schematic of self-assembly of nanoarchitectures from Janus dendrimers and the corresponding structure–concentration phase diagram for amphiphilic dendrimer aqueous solutions [150]. (**b**) Self-assembled morphologies by various C_a_-POSS_b_-PEG_c_ polymers observed by microscopy (**top**, with zoom-in on nanoscopic feature in the middle panel) and corresponding DPD modelling images of self-assemblies by the amphiphilic polymers at the initial polymer concentration of 1 mg/mL in aqueous solutions (**bottom**) [153]. (**c**) Typical calculated states of the interactions between a lipid bilayer membrane and a PAMAM dendrimer conjugated with ligands. The receptor density of the membrane is fixed at f_R_ = 0.33. The surface tension of the membrane, σ, and the ligand density of the dendrimer, f_L_, are (**top left**) σ = 2.09 k_B_T/r_c_^2^ and f_L_ = 0.1, (**top right**) σ = 0.10 k_B_T/r_c_^2^ and f_L_ = 0.4, (**bottom left**) σ = −0.65 k_B_T/r_c_^2^ and f_L_ = 0.6, and (**bottom right**) σ = 1.10 k_B_T/r_c_^2^ and f_L_ = 0.8. Color scheme: lipid head beads (pink), lipid tail beads (cyan), receptor beads (green), charged beads of dendrimer (red), uncharged beads of dendrimer (yellow), and ligand beads (blue) [156].

Hu et al. [150] investigated the formation mechanism of dendrimersomes through MD simulations of the formation of synthetic vesicles from amphiphilic Janus (two-sided) dendrimers. They created the spherical single-site Janus particle using an anisotropic potential to mimic the two distinct surfaces, one hydrophobic side and another hydrophilic side (see Figure 4a). This simple model allowed them to model both the concentration-dependent growth of structures, both the enthalpy-mediated formation process of onion-like dendrimersomes and the alternative entropy-mediated self-assembly of amphiphilic flexible chains. Linear micelles, lamellar structures and vesicles were observed in the simulations (see Figure 4a). They also found that the size of dendrimersomes will not increase through mutual fusion once the well-defined onion-like structure is formed, unlike numerous lipidsomes and polymersomes that can spontaneously coalesce. Future work combining their hard chain model with the previous reports using flexible chain models [144,147,148,149,151,152] could more fully characterize and predict the potential of dendrimersomes for applications in drug and gene delivery.

Wang et al. [153] reported a combined experiment–simulation co-study detailing a general strategy to construct uniform aggregates by manipulating self-assembly of dendrimers with precisely controlled polyhedral oligomeric silsesquioxane (POSS)-embedded cores. The authors created different types of amphiphilic dendrimers with rigid–flexible coupling POSS-embedded cores, different PEG chain lengths, and various geometries. They found that the rigid POSS molecules could grow in ordered arrangements while the flexible alkyl chains could rearrange to minimize the free energy of the assembly. PEG chains with extended conformations influenced the configuration of the inner core by occupying the excluded volume of the hydrophobic regions. DPD simulations substantiated their experimental findings, allowing better understanding of the underlying self-assembly processes. As an example, they studied the self-assembly of amphiphilic C3-POSS3-(PEG550)*x* polymers with different hydrophobic/hydrophilic ratios created by simply changing the PEG length *x*. With increasing hydrophobicity, spherical micelles, rod micelles, and vesicles were obtained from DPD simulations in good agreement with the images obtained from electron microscopy (see Figure 4b). The results obtained in this work provide a methodology to broaden the variety of rigid–flexible core dendrimers to fabricate responsive hierarchical self-assemblies for biomaterial science and biomimetic nanotechnology [154,155].

Combining DPD simulation and analytic models, Yan et al. [156,157] studied the molecular mechanism and kinetic behavior of the transmembrane transport of PAMAM dendrimer-like soft NPs conjugated with ligands. They identified three different states during the interaction between the dendrimer and the cell membrane: penetration, partial wrapping, and full wrapping (see Figure 4c). Both the membrane tension and the ligand density on the NPs influenced all three stages.

### 2.2. Polyelectrolytes

Polymers carrying a net negative or positive charge (see Figure 1d) at or near neutral pH are called polyelectrolytes (PEs). Their solubility in water is driven by electrostatic interactions between water and the charged monomer [158]. Examples of such polymers include DNA, protein and some derivatives of cellulose polymers. PEs are classified according to four main categories: natural/synthetic, homopolymers/copolymers, linear/branched/cross-linked and polyanions/polycations/polyampholytes [159]. Many researchers have extensively investigated the properties of the individual polyelectrolytes and the formation of polyelectrolyte complexes (PECs) [160,161]. The structures created by the collapse of PEs by, for example, macro-ion like proteins [162], ionic surfactants [163], or oppositely charged PEs [164,165] are usually described as a “polyelectrolytes complex”, which has wide applications in functional nanomaterials, gene therapy, and drug delivery [166,167,168,169,170,171]. The formation mechanism of a polyelectrolyte complex can be explained by theories based on the electrostatic forces and Flory–Huggins mixing free energies of the polyelectrolytes [172,173]. When the charge fraction of the chains is low, the polymer backbone repulsion (Flory–Huggins interaction parameter) is dominant and the solution separates into two phases, each containing mostly one of the polymers, while at high charge fraction, the attractive electrostatic interactions between the polymers is the controlling factor which stimulates precipitation and formation of a complex [174,175].

The concept of PECs in the design of drug delivery systems may be useful because they can alter drug physicochemical properties such as stability and dissolution [171,176,177,178]. The drug molecules can be encapsulated through incorporation into PECs by being: (1) entrapped from the solution during precipitation of the complex, (2) absorbed on the already formed complex, (3) chemically bonded to at least one partner, or (4) itself a charged partner for PEC [179]. Polyelectrolyte–drug complexes have an amorphous colloidal structure that may be suited for delivery of poorly soluble drugs [180,181,182,183]. An example of a clinical study on a polyelectrolyte-based drug delivery system is presented in Table 2. The formation of the PECs is a spontaneous reaction which appears in aqueous environments at the stage of polymer mixing, yet its complex nature stems from the dual character of macromolecules and electrolytes. This may, in turn, cause difficulties in providing the structural homogeneity that may aid their deployment in living systems. A number of technological factors, including pH alterations, charge density, or polymer concentration, may determine or modulate the physicochemical and biological properties of the PECs formed [184]. Upon ionization, polyelectrolytes become pH-responsive polymers that can undergo various changes in their physical structure, including extension of their coiled chains as a result of electrostatic repulsion between positive charges generated protonation at low pH [185]. Various physiochemical characteristics of polyelectrolytes including solubility, chain conformation, self-assembly of polymers/copolymers, aggregation size, shapes and volume of the individual counterparts can be adjusted by controlling the pH level [97]. Polyelectrolyte NPs are often polydisperse systems with broad size distributions ranging between 10 nm and 1 µm [186]. pH-responsive behavior of polyelectrolytes has been used to induce the controlled release of model compounds such as vitamin B12 [187] and drugs including ibuprofen [188].

**Figure 5:**
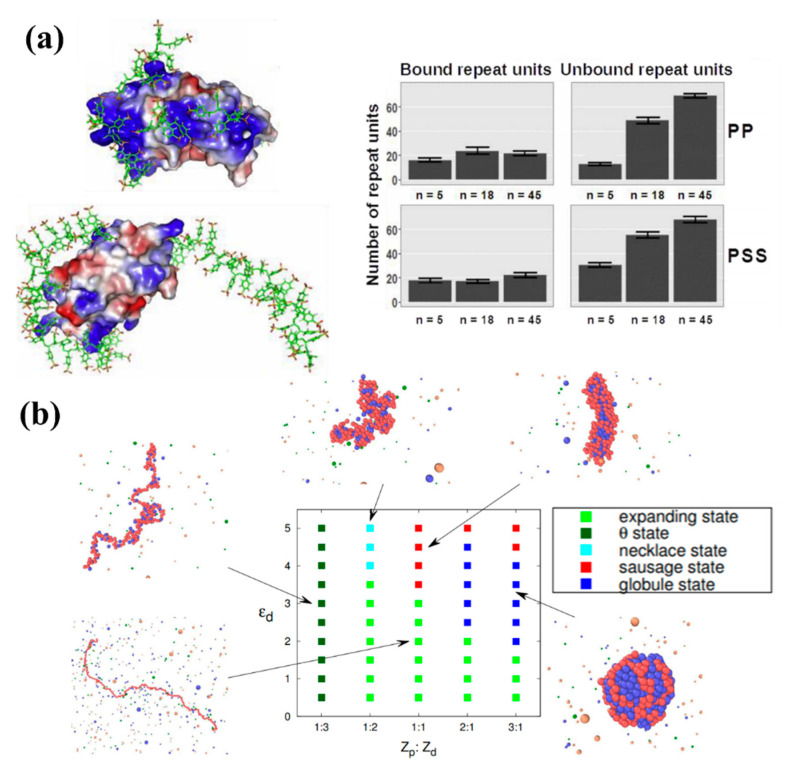
(**a**) Computed protein–carrier structures made from poly(styrene sulfonate) (PSS) and polyphosphate (PP), showing PSS5 (**top left**) and protein-PSS45 (**top right**) complexes. The polyanion is shown in sticks, the protein is shown in surface representation and colored according to electrostatic potential. The number of bound and unbound repeat units of PP (**middle**) or PSS (**bottom**) chains (n is a degree of polymerization) is plotted underneath [191]. (**b**) Morphological phase diagram of the PE–drug complexes characterized using drug hydrophobicity ε_d_ and PE-drug valence ratio Z_p_:Z_d_. [192].

Despite the large amount of research works on the behavior of polyelectrolyte chains, the size and complexity of the assemblies has limited modelling of the polyelectrolytes-drug complex, in which the polyelectrolyte chains and opposite charged hydrophobic drug molecules form a complex that can protect the drugs [189,190]. As an example, Sofronova et al. [191] studied the influence of degree of polymerization on the structure and properties of the formed soluble protein–polyelectrolyte complexes. Using MD simulations, they modelled the structure, dynamics and energetics of complexes of cationic protein lysozyme with highly charged polyanions poly(styrene sulfonate) and polyphosphate created with different degrees of polymerization. The computed complexes revealed that the short charged chains efficiently coated the protein, while the long chains interacted with the protein mainly through the charged loops and tails (see Figure 5a), keeping the proteins in their native state and protecting against aggregation. Given this ability to make stable, specific complexes between PEs and naturally charged macromolecules such as proteins, one potential application would be the design of platforms for antibody drug delivery systems that protect against damage during different administration methods. Modelling results such as those provided by Sofronova et al. can give a general perspective on the influence of polymer type, length and charge on the mechanism of assembly of the desired polyelectrolyte-based protein delivery system.

Lei et al. [192] combined mean-field theory and extensive molecular simulations to study the phase behavior of the PE–drug complex in dilute, salt-free solution. They focused on the morphologies of the complexes with varying drug hydrophobicity and different PE–drug valence ratios. They obtained a phase diagram in which five different main morphologies including the expanding state, the θ condition state (in which the chain behaves exactly as predicted by the random walk or ideal chain model), the necklace state, the sausage state and the compact globular state were identified (see Figure 5b). The benefit of this phase diagram is the provided broad information on PE–drug complex response to drug hydrophobicity and PE–drug valence ratios, which can be used in both drug encapsulation and release processes. They found that the complexation is a first-order-like phase transition controlled by the hydrophobic attraction between the drug molecules. They also predicted that the stability and morphology of the complex was determined by the valence ratio between the drug molecule and PE monomer. Finally, by exploring the dynamics aspect of PE–drug complexation, they found that extremely hydrophobic drug molecules could trap the complex in a non-equilibrium glass-like state. Their work can provide guidelines to fabricate colloidal PE–drug complexes with “dialed-in” desirable physical characteristics.

### 2.3. Cyclic Copolymers

#### 2.3.1. Overview

Cyclic polymer-based structures are challenging to synthesise and purify but show good potential as drug nanocarriers due to their special architecture and stimuli responsive behaviors [193,194,195,196,197,198,199,200,201]. The relative size of the resulting copolymer assemblies is influenced by the conformation of the different architectures. As the core-forming block of the cyclic diblock copolymer assembly is required to loop and cannot stretch without restriction, the value of radius of hydration (*R*_h_) for a cyclic diblock micellar assembly is expected to be larger than that of the equivalent linear diblock for a given block composition (for example, for cyclic-PEO42-*b*-PBO8 *R*_h_ = 4.4 nm, while for linear PEO21-*b*-PBO8-*b*-PEO21 *R*_h_ = 4.0 nm) [202]. Zhang et al. [203] reported that the hydrodynamic diameter of cyclic poly(ethylene glycol)-b-poly(ε-caprolactone) (PEGx-b-PCLy) micelles was approximately half that of linear PEGx-b-PCLy micelles.

Different cyclic polymer materials are designed to be responsive to different stimuli such as photonic, thermal, electronic or chemical [201]. For example, polystyrene [204], poly(methyl methacrylate) [205] or polythiophene [206] derivatives can make photo responses in cyclic polymers. Thermo/chemo responses can take place via poly(aldehyde) [207], and cyclic–linear topological transformation that can be triggered by poly(ethylene oxide) (or polyethylene glycol) [207]. Additionally, in biomedical fields, the topology effects of cyclic polymers was exploited to achieve controlled/improved biotransportation as well as gene/DNA delivery [201]. For the former application, poly(acrylic acid) derivatives grafted to polyethylene glycol [208] or oligopeptides [209] were incorporated into the cyclic polymer structure, and for delivery, cationic derivatives of poly(methyl methacrylate) [210] and polyethylene imine were used [211].

In the following, we discuss some of the most recent and promising predictive modelling-based research that can guide future experimental efforts to self-assemble functional cyclic polymer-based nanostructures.

Liu et al. [212] combined DPD with all-atom MD simulations based on the ABEEM (atom-bond electronegativity equalization fluctuating charge force field model) polarizable force field to study the self-assembly of linear and cyclic polystyrene (PS)-polyisoprene (PI) di-block copolymers, PS_290_-PI_110_, in n-heptane. This work was the first try to combine DPD simulations and all-atom MD simulations based on a polarizable force field, in an effort to quantify the effect of architecture and blending on the self-assembly properties in solution. The ABEEM polarizable force field provides a more accurate treatment of the intermolecular interactions in the system than traditional nonpolarizable force fields [213]. Their results demonstrate that the combination of DPD and MD with a polarizable force field can efficiently bridge the gap between atomistic and mesoscopic simulations, and enables the accessing of larger length scales and longer time scales while preserving atomic scale detail. By comparing the self-assembly behavior of cyclic di-block copolymers with those of their analogous linear block copolymers, they found the PS-PI cyclic block copolymers self-assembled into cylindrical micelles, while spherical micelles with stable structures formed from the linear PS-PI block copolymer with the same composition. In both structures, the low-polarity PS blocks were distributed inside the micelle, forming a hydrophobic core, and the high-polarity PI blocks spread around the surface, forming a protective shell. According to their results, the self-assembled morphologies could be changed dramatically by the addition of PS homopolymers; for cyclic copolymer from cylindrical micelles to vesicle, and for the linear copolymer from spherical to cylindrical micelles

The self-assembly of microstructures from amphiphilic cyclic brush-copolymers in solution was investigated by Yang [214] using DPD simulations. Cyclic brush copolymers are innovative materials with a cyclic core hosting radiating polymer brushes producing myriad polymer topologies. The authors could obtain a series of self-assembled structures, such as rods, plates, vesicles, large compound vesicles, bilayers, and spheres from the solutions by changing solvophilic/solvophobic side chain lengths, solvophilic/solvophobic backbone lengths, and grafting densities. For example, in the case of vesicle structure, they found that increasing the solvophobic side chain length or solvophobic backbone length decreases the cavity size and increases the membrane thickness, while the whole vesicle sizes remained near-constant. They also pointed out that self-assembly of a plate structure with larger thickness and narrower width required increased solvophobic side chain or backbone lengths. Their most useful finding was that amphiphilic cyclic brush copolymers with higher backbone asymmetry and larger grafting density can form morphologies with more curved interfaces. This study can provide valuable guidance to design cyclic brush copoloymers, as a complex functional material, to form different self-assembly structures that could be useful in many applications including drug delivery, bioimaging and nano- or microreactors.

#### 2.3.2. The Informative Representative Example of Cyclodextrin

One of the remarkable cyclic polymers with exceptional properties is cyclodextrin (CD) [215,216] that has high potential as a promising delivery platform for therapeutic oligonucleotides [217,218,219] (see Table 2 to find some clinical studies on the CD based drug delivery system). Cyclodextrins are natural cyclic oligosaccharides composed of six (α-CD), seven (β-CD) or eight (γ-CD) glucopyranoside units linked by α-1,4-glycosidic bonds [220], with hydrophilic primary and secondary faces and a hydrophobic cavity. The hydroxyl groups on the ring structure provide the opportunity to functionalize and provide amphiphilic, cationic, anionic, PEGylated and targeted CDs [217,221]. Herein, we review the recent modeling research works on CDs self-assembly and interactions with drug molecules.

Zheng et al. [222], investigated the host–guest interaction between α-CD and azobenzene-containing amphiphile 1-[10-(4-phenylazophenoxy)decyl]pyridinium bromide (AzoC10), which is a photoresponsive material and can undergo *cis–trans* photoisomerization in response to UV and visible light. They used coarse-grained molecular dynamics (CGMD) simulations with the modified MARTINI force field to investigate the assembly of *cis*-, *trans*-AzoC10, and *cis*-, *trans*-AzoC10/*α*-CD into micelles in water. By analyzing the size and shape of spontaneously assembled micelles, they realized that the shape of the obtained aggregate depended on both the molecular structure and the monomer concentration in the following way: both *cis*- and *trans*-AzoC10 aggregated into spherical micelles at low concentrations, while at high concentrations, *cis*-AzoC10 showed co-existing disk-like and spherical micelles but *trans*-AzoC10 formed co-existing worm-like and spherical micelles. In mapping the dynamics of the aggregation, the authors divided the self-assembly process into three stages: rapid nucleation; formation and growth of spherical micelles; and formation of disk-like or worm-like micelles. In the self-assembly of *cis*-AzoC10/*α*-CD, the hydrophobic azobenzene moieties aggregated to form the inner core of worm-like micelles with outward-pointing hydrophilic pyridinium head groups. The worm-like micelles were surrounded by α-CDs to shield the hydrophobic azobenzene group against water. However, due to the bulky size of α-CD, some hydrophobic azobenzene groups were still exposed and loosely packed. According to their results, *cis*-AzoC10/*α*-CD aggregated into worm-like micelles at all concentrations, while in *trans*-AzoC10/*α*-CD, the loss of amphiphilicity caused by axial configuration of the hydrophobic azobenzene moiety in the *α*-CD cavity [223] destabilized the micelle structure.

**Figure 6:**
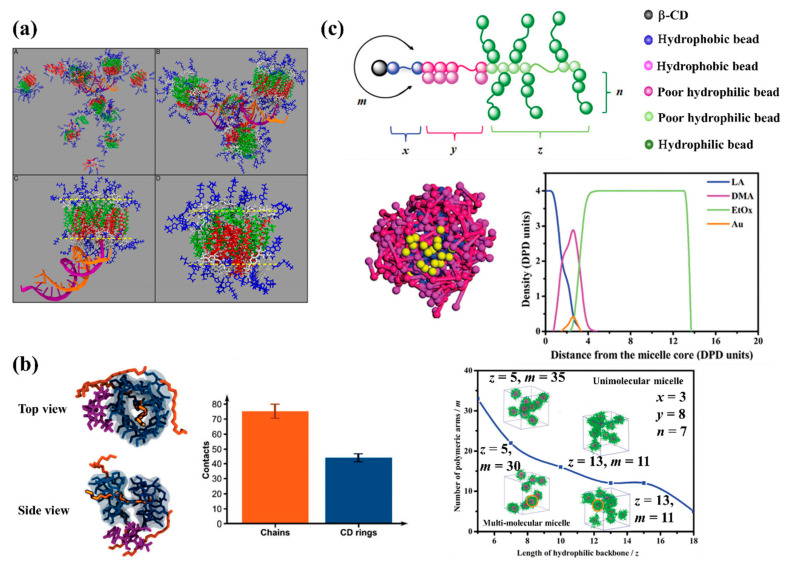
(**a**) Representative computed self-assembled multimeric units of 39 cationic functionalized CD molecules around siRNA [224]. (**b**) Computed di-oleoyl-glycerolipidyl-β-cyclodextrin–atazanavir (DOCD–ATAZ) (2:1) complex after 100 ns of MD simulation (**left**) (**top** and side views) (ATAZ is represented in purple) and (**right**) average number of contacts between DOCD moieties (lipid chains in orange and the CD moiety in blue) and ATAZ atoms for the MD simulation of 36 DOCD with 18 ATAZ [225]. (**c**) Coarse-grained models of the β-CD-copolymer (**top**), computed cross-section views of β-CD-copolymer/gold nanoparticles at 1:3 molar ratio, corresponding density profiles of different layers (**middle right**) and morphologies of micelles assembled with different building-block topologies (**bottom**) [226].

Singh et al. [224] examined the dynamical self-assembly behavior of cation-functionalized β-cyclodextrin (CD) derivatives around siRNA using classical MD simulations in water with physiological salt ionic strength. They found the cationic CD molecules spontaneously formed superstructures in solution, which assembled around siRNA to form a stable host that stabilized siRNA via electrostatic interactions. They concluded that the superstructures formed by the cationic CD molecules constitute an ideal platform to encapsulate negatively charged siRNA molecules, which could provide a promising gene delivery vector (and in future work, delivery of negatively charged proteins or other macromolecular drugs). The cation CD lipid-like behavior in solution enabled creation of stable superstructures (see Figure 6a), providing nanoscale molecular templates with highly controllable size and shape. Due to their amphiphilicity and ability to assemble into encapsulating superstructures, these types of self-assembled CD-based carriers can boost membrane permeability and so improve transfection.

Furlan et al. [225] used MD simulation-guided experiments to study lipid-functionalized β-CD (di-oleoyl-glycerolipidyl-β-cyclodextrin, DOCD) self-assembly and drug encapsulation in the DOCD formed NPs. MD simulations predicted entrapment of atazanavir (ATAZ), as the drug model, in the NPs of DOCD. In order to investigate DOCD–ATAZ interactions, they implemented two types of MD simulation. The first one was a long simulation of a system which contained two DOCD molecules with one ATAZ to examine whether a complex of DOCD/ATAZ could form. The results showed that the ATAZ molecule was not placed inside any of the two hydrophobic cavities (see Figure 6b), but rather became entrapped in lipid chains. In the second type of MD simulation, they evaluated the potential changes in DOCD aggregation due to the presence of ATAZ, through simulation of a larger model composed of 36 DOCD and 18 ATAZ molecules. Their results confirmed that ATAZ molecules did not preferentially bind in the hydrophobic CD cavity but remained in close contact with the CD moieties through side interactions (see Figure 6b). They concluded that ATAZ molecules were more likely to interact with the DOCD lipid moieties than with CD rings, based on the larger number of contacts between ATAZ and DOCD lipid chains than between ATAZ and the CD macrocycle.

Zhang et al. [226] performed DPD simulations to study the formation process of unimolecular micelles from a β-CD-based star-like architecture created using a complex organic block co-polymer (poly(lactide)-*block*-poly(2-(dimethylamino) ethyl multimethacrylate)-*block*-poly[oligo(2-ethyl-2-oxazoline)methacrylate)(PLA-*b*-PDMAEMA-*b*-PEtOxMA). Their results predicted the formation of thermodynamically stabilized unimolecular micelles, which could stay as unimolecular micelles within a certain concentration range, indicating their potential to maintain a constant particle size and a stable structure in vivo. This would be a useful feature of an efficient drug transporter material. To prepare unimolecular micelles, a reasonable design of star-like copolymer is required. Parameters like hydrophobic or pH-sensitive chains length, shorter hydrophilic backbones length or hydrophilic side chain grafting density, and number of polymer arms, can shift the hydrophilic–lipophilic interaction balance, and non-prudent material choices can easily disrupt the formation of unimolecular micelles. The authors further examined encapsulation of small gold nanoparticles into the β-CD-based polymeric unimolecular micelles, with DPD simulations revealing that Au nanoparticles tended to distribute only in the middle PDMAEMA layer.

## 3. Conclusions

Here we have reviewed the latest knowledge available on three types of polymers with unusual architectures including dendritic polymers, polyelectrolytes and cyclic polymers with a focus on the potential applications of their self-assembled NPs in drug delivery. Ideally, stimuli responses, drug encapsulation/release, and carrier disassembly mechanisms should be encoded at the molecular level. Let us compare the self-assembly mechanisms of the three reviewed NP platforms: Drugs can be encapsulated by dendritic polymers through adsorption/conjugation on the polymer surface or penetrate into the voids, which are both enthalpy driven processes (van der Waals and (or) electrostatic driven), by contrast, the main mechanism of drug encapsulation in polyelectrolyte NPs is complex formation which is electrostatically driven, and in cyclic polymer NPs, drugs can be encapsulated in the core of the formed micelles through an enthalpy driven process. However, it should be emphasized that the size and shape of the final formed NPs are the direct consequence of the architecture of the polymers (entropy contribution) plus their chemical composition (enthalpy contribution). As discussed above, NP chemical compositions can be tunable for the desired drug and the administration method, by functionalization of the polymers with different charged/neutral functional groups/blocks. There are different parameters related to shape and charge of these polymers that can individually or collectively direct the encapsulation mechanism, stimuli responses and disassembly mechanism. This can be achieved by changing enthalpy or entropy contribution significance through turning the following structural parameters: (1) generation, terminal groups, flexibility, surface charge, length of arms, number of arms, and surface tension for dendritic polymers; (2) ionic strength, chain flexibility, backbone hydrophobicity and degree of polymerization for polyelectrolytes, and (3) degree of polymerization of backbone, backbone flexibility, hydrophobic/hydrophilic block length ratio, branch length (for brushed cyclic polymers), hydrophobic/hydrophilic branch length ratio, backbone or branch (for brushed cyclic polymers) functionalization for cyclic polymers. Understanding the effects of these parameters in NP-mediated drug delivery can inform rational design of smart multifunctional polymer-based NPs drug delivery systems. We summarized the achievements of some recent research studies related to the effect of some of the above-mentioned parameters in Table 3.

## 4. Future Outlook

In this focused review article, we summarized and discussed the latest exciting developments in the self-assembly of smart multifunctional polymer-based nanoparticles (NPs), through the lens of advances in predictive modelling-led design. We have identified some important challenges and opportunities for further progress, and we hope that our perspective will stimulate more modelling-guided investigations into the synthesis, self-assembly, and applications of smart polymer-based NPs. In our opinion, the future of the self-assembly of smart polymer-based NPs lies in:

(1) **Screening studies with new polymer compositions**: Construction of smart polymer-based NPs with rationally selected new polymer compositions. This can be done using copolymerization or post-synthesis modification. The so-called functional monomers can be conjugated molecules, biomolecules, or inorganic nanostructures. In this way, the obtained self-assembled NPs with new compositions may self-assemble into some new types of superstructures and morphologies with potentially useful functions.
(2) **Hybrid organic–inorganic materials**: Self-assembled hybrid organic and inorganic smart NPs may provide a new generation of nanocarriers that interact controllably with biological material while exhibiting also useful electronic, magnetic and optical properties.
(3) **Multiphasic systems**: The scope of smart polymer-based NPs shall be expanded from the binary system to ternary or even multicomponent system by improving the types of monomers that take part in the click polymerization simultaneously. Appropriately parametrized and benchmarked high-throughput computational screens could rapidly accelerate this process.
(4) **Multifunctional architectures**: Employing smart multifunctional copolymer/polymer combinations may provide new types of architecture for smart drug delivery-based NPs, which can be engineered by changing parameters related to the relative concentration, size, composition and chemistry of the components, molecular symmetry, and relative flexibility/mechanical/thermal properties of the components. All the effective parameters governing the self-assembly can be examined via multi-scale molecular simulations to provide a comprehensive guide to “shortest path” experiments.
(5) **Expansion of molecular simulation protocols towards accelerated materials screening**: This would allow reliable prediction of the self-assembly behavior of smart drug delivery-based NPs, utilizing emerging methods that can rapidly explore large space–time scales [227].
(6) **Other applications besides drug delivery**: Taking full advantage of smart NPs self-assemblies to employ their unique characteristics in other applications, such as polymerization, protective coatings and miniaturized electronic devices.

In summary, the self-assembly of smart polymer-based NPs is a nascent, rapidly developing field. We believe that focused fundamental research will create immense opportunities for their deployment, particularly in the development of atomistically-benchmarked simulation methods that can approach micrometer length and millisecond time scales.

## Figures and Tables

**Table 1:** General descriptions of the common simulation methods applied for drug-delivery studies.

Scales	Length and Time Scales [74]	Descriptions	Formulations
Quantum scale	~10^−10^ m and ~10^−12^ s	The nuclei and electrons are the particles of interest at this scale and quantum mechanics (QM) methods are used to model their state by solving the Schrödinger wave equation [74].	$-\frac{h^{2}}{8\pi^{2}m}\nabla^{2}\phi {\left(r\right)}_{k}+U\left(r\right)\phi {\left(r\right)}_{k}=E_{k}\phi {\left(r\right)}_{k},$ $\phi {\left(r\right)}_{k}:\mathrm{Wave}\;\mathrm{equation}$ *E_k_*: energy eigenstate U(r): Potential *h*: Planck constant *r*: coordinates vector *m*: mass
Atomistic scale	~10^−9^ m, ~10^−9^–10^−6^ s	The Monte Carlo (MC) technique is a stochastic method that uses random numbers to generate a sample population of the system from which one can calculate the properties of interest [74,75].	A new configuration can be produced by arbitrarily or systematically moving one atom from position *i* →j and can be accepted if ∆H=H(j)−H(i)<0 If ∆H>0 the move is accepted only with a certain probability $p_{i\to j}$ which is given by $p_{i\to j}\propto \exp\left(-\frac{∆H}{k_{B}T}\right)$. According to Metropolis et al. [76], one can determine the new configuration according to the following rule: $\xi \leq \exp\left(-\frac{∆H}{k_{B}T}\right),\;\mathrm{the}\;\mathrm{move}\;\mathrm{is}\;\mathrm{accepted};$ $\xi >\exp\left(-\frac{∆H}{k_{B}T}\right),\;\mathrm{the}\;\mathrm{move}\;\mathrm{is}\;\mathrm{not}\;\mathrm{accepted}.$ *H*: Hamiltonian *k_B_*: the Boltzmann constant ξ: a random number between 0 and 1
		The Molecular dynamics (MD) simulation technique allows one to predict the time evolution of a system of interacting particles (e.g., atoms, molecules, granules, etc.) and estimate the relevant physical properties [75].	The simulation of a many-body system would require the formulation and solution of equations of motion of all constituting particles, which for a particle *i* is $m_{i}\frac{d^{2}r_{i}}{dt^{2}}=f_{i}$, *m_i_*: the particle mass ***r**_i_*: the particle position vector. ***f**_i_*: the force acting on the *i*th particle The interaction potentials describe in detail how the particles in a system interact with each other, i.e., how the potential energy of a system depends on the particle coordinates. Some of the most common simulations use AMBER [77], GROMOS [78] CHARMM [79] and OPLS [80].
		Molecular mechanics (MM) is a simulation technique to minimize large molecular structures such as DNA, RNA, proteins and their complexes, in which atoms are treated as masses, and bonds as springs with appropriate force constants. For minimizations calculations, the positions of the atoms within a molecule must be systematically or randomly moved and the energy recalculated with the goal of finding a lower energy and hence more stable molecule [81,82].	Similar to MD simulation, MM is based on Newton’s equation of motion. The interactions between the particles in the system can be described via the force-field potentials applied in MD simulations [82].
Mesoscopic scale	~10^−6^ m, ~10^−6^–10^−3^ s	Coarse-grained molecular dynamics (CGMD) methods overcome length and time scale limitations of atomistic simulations though coarse-graining large molecules by several connected beads [13].	Commonly used forcefields in CGMD are: Weeks–Chandler–Andersen potential, COS potential and Finite Extensible Elastic (FENE) bond potential [83]. MARTINI forcefileds [70].
		The Dissipative particle dynamics (DPD) method is also a mesoscopic simulation technique which can correctly account for the hydrodynamic interactions by considering water molecules explicitly. In DPD simulations, a cluster of atoms are represented by one bead and its dynamics is governed by Newton’s equation of motion [13,74,75].	Beads *i* and *j* interact through simple pairwise force consisting of a conservative force (*F^C^_i_*_j_), a dissipative force (*F^D^_i j_*), and a random force (*F^R^_i j_*). The total force applied on each bead *i* due to bead *j* is given as a sum of these three terms [71] $F=F_{ij}^{C}+F_{ij}^{D}+F_{ij}^{R}$

**Table 2:** Clinical studies on the reviewed smart nanoparticles (NPs).

Delivery System Platform	Nanoparticles	Clinical Study
Dendritic polymers	DEP™-Docetaxel DTX-SPL8783 [128] (DEP dendrimer with docetaxel and PEG terminal blocks)	Advanced or metastatic cancer
	VivaGel^®^ SPL7013 [128] (an active ingredient is a generation 4 lysine dendrimer, ended by a 2-[(3,6-disulfo-1-naphthalenyl)oxy] acetic acid disodium salt)	Bacterial vaginosis
	Hydroxyl-terminated PAMAM dendrimers [129]	Neuroinflammation in a large animal
Polyelectrolytes	Protamines [130]	Approved by the FDA for clinical applications, including insulin delivery and reverting heparin-induced anticoagulation.
Cyclic polymers	An anticancer agent CALAA01, a targeted, self-assembling nanoparticles system based on CD complexed siRNA [131] Piroxicam-beta-Cyclodextrin [132] Nimesulide-beta-Cyclodextrin [132] Aceclofenac-beta-Cyclodextrin [132]	In phase I clinical trials for the treatment of solid tumours Anti-inflammation Anti-inflammation Anti-inflammation

**Table 3:** Summary of the molecular shape and charge effects on the NPs morphology for drug delivery application.

	Molecular Features Effect		Simulation Method	Refs.
	Molecular Shape/Property	Charge		
Dendritic polymers	Flexibility: The more flexible dendrimer architecture could achieve more conformational rearrangement of its surface functional groups to optimize induced-fit with DNA.		MD	[143]
	Generation: Large charged dendrimers have more charges which would strengthen the rigidity of the dendrimers through electrostatic repulsion, resulting in a less induced-fit with DNA.	Charge: The rigidity of the dendrimers with charged functionalized groups on their surface is higher than neutral ones, which affects the self-assembly structure.	DPD	[134]
	Surface branch length: The lower hydrophilic branches are in an amphiphilic hyperbranched copolymer, formation of membranes/vesicles/vesosomes-shaped nanoparticles can be more probable by tuning the solvent selectivity of the polymer blocks, while increasing the branches’ lengths provide the chance of small micellar aggregates formation.		DPD	[148]
	Surface branch length in a rigid-core dendrimer: With increases in the branches’ lengths (decreasing hydrophobicity), vesicle, rod micelles and spherical micelles can form.		DPD	[152]
Polyelectrolytes	Degree of polymerization: In a polyelectrolyte/protein complex the short charged chains efficiently coated the protein, while the long chains interacted with the protein mainly through the charged loops and tails, keeping the proteins in their native state and protecting against aggregation.		MD	[191]
		Charge: To encapsulate hydrophobic drug molecules in an efficient globular polyelectrolyte-drug complex structure, a high monomer/drug charge ratio is required.	CGMD	[192]
Cyclic polymers	Hydrophobicity/Hydrophilicity: In a cyclic copolymer, self-assembled morphologies could be changed from cylindrical micelles to vesicle by increasing hydrophobicity/hydrophilicity (hydrophobic block length/ hydrophilic block length).		DPD	[200]
	Surface branch length: In a cyclic brush copolymer, by increasing the hydrophilic branches length, regular core-shell micelles from. By increasing hydrophobic branch length to protect the hydrophobic blocks against solvents, plate-like structures or vesicles can be observed, as well as the final self-assembled morphology.		DPD	[213]

## Data Availability

Data available in a publicly accessible repository.
